# Whole-Word Phonological Representations of Disyllabic Words in the Chinese Lexicon: Data From Acquired Dyslexia

**DOI:** 10.1155/2005/597581

**Published:** 2006-01-09

**Authors:** Sam-Po Law, Winsy Wong, Karen M. Y. Chiu

**Affiliations:** ^1^Division of Speech and Hearing SciencesThe University of Hong KongPokfulam RoadHong Kong SARChina; ^2^Tung Wah HospitalHong KongChina

## Abstract

This study addresses the issue of the existence of whole-word phonological representations of disyllabic and multisyllabic words in the Chinese mental lexicon. A Cantonese brain-injured dyslexic individual with semantic deficits, YKM, was assessed on his abilities to read aloud and to comprehend disyllabic words containing homographic heterophonous characters, the pronunciation of which can only be disambiguated in word context. Superior performance on reading to comprehension was found. YKM could produce the target phonological forms without understanding the words. The dissociation is taken as evidence for whole-word representations for these words at the phonological level. The claim is consistent with previous account for discrepancy of the frequencies of tonal errors between reading aloud and object naming in Cantonese reported of another case study of similar deficits. Theoretical arguments for whole-word form representations for all multisyllabic Chinese words are also discussed.

## 1. Introduction

In the past several decades, the recognition and production of multimorphemic words have attracted much attention in psycholinguistic research. These observations inform us about their representations and the architecture of the mental lexicon. For (highly) inflected languages, several approaches have been proposed. The morpheme-based approach suggests that the units of access are individual morphemes and morphologically complex words are automatically decomposed (or affix-stripping) during processing. A representative model of this type contends that lexical access takes place via an entry representing the stem of the stimulus. Information on possible affixation to the stem is stored within that lexical entry [17,18]. In contrast, the whole-word representation approach claims that multimorphemic words are stored as wholes. They are the only units for lexical access, and they may or may not be marked morphologically (e.g. [12,15]). Another type of models, illustrated by the Augmented Addressed Morphology (AAM) model, assumes that the lexicon contains both whole-word and morpheme units [1,3]. The latter represent stems and affixes at the same level. Stems and affixes are connected with each other if they form a real word, i.e. a morpheme network. The recognition of an existing multimorphemic word involves the activation of units corresponding to the whole word as well as the constituent morphemes. On the other hand, novel words only access morpheme units.

In more recent years, questions have been raised as to whether these models can shed light on the representation and processing of morphologically complex words in languages with little inflectional morphology, such as Chinese. There are few affixes, derivational or inflectional, in this language. The great majority of morphemes are "stem" morphemes corresponding to single syllables and single characters. Compounding is extremely productive. In other words, most Chinese words are disyllabic or multisyllabic. Other mul-

ISSN 0953-4180/05/$17.00 © 2005 – IOS Press and the authors. All rights reserved tisyllabic words are binding words whose constituents must co-occur and very rarely combine with other morphemes to form lexical items (e.g. 蚯蚓 *jau1 jan5*[1] 'earthworm', 徘徊 *pui4 wui4* 'to linger'), loanwords (e.g. 玫瑰 *mui4 gwai3* 'rose', 葡萄 *pou4 tou4* 'grapes'), and phonetic transliteration of foreign items (e.g. 杯葛 *bui1 got3* 'boycott', 幽默 *jau1 mak6* 'humor').

Theories of the Chinese mental lexicon have essentially been developed based on data from normal subjects on reading aloud and lexical decision latencies of single characters and compound words. In earlier models, whole words and morphemes were represented at separate levels in a hierarchical relationship. In the multilevel interactive-activation model [20, 32], there were modality-specific morpheme and word units. When the system was presented with a compound, the relevant units at the morpheme level would be activated first; activation would then pass up to the word level followed by the semantic level. Connections between units at different levels are excitatory, whereas those between units at the same level are inhibitory. Evidence was also provided to argue for linkage between phonological and orthographic units at the morpheme level [20] and the word level [21]. The multilevel cluster representation model for spoken word processing [26–29] recognized three levels of representations, i.e. syllable, morpheme, and word levels. Units at the morphemic and word layers were organized in clusters on phonological grounds. That is, homophonous morphemes and words sharing the first syllable formed clusters at the morpheme and word levels, respectively. Representations in the same cluster competed with each other in word recognition. Although there were no direct connections among words having the same morpheme, they were indirectly linked via their connections with units at the morpheme layer. The presence of whole-word representations of compounds was motivated by the findings that lexical decision response time was mainly determined by word frequency, which did not interact with morpheme or syllable frequency of either of the constituent morphemes in semantically transparent compounds [28]. A word is semantically transparent if its meaning can be derived from the meanings of its constituents, e.g. *pei4 haai4* leather-shoe 'leather shoes'.

More recent versions of these models have converged on an architecture with two basic characteristics: (i) there are three types of units, semantic features, single syllables and single characters, that are interconnected;[2] (ii) whole-word and morphemic representations are purely semantic in nature and their relationship is not hierarchical [19,30,31]. The former purports to reduce the redundancy in representation at the phonological and orthographic levels, as the majority of multisyllabic or multicharacter words are simply the concatenation of their constituents. The latter was motivated by the observations that priming effects found in lexical decision tasks were largely meaning-based. More specifically, Taft et al. [19] reported that semantically opaque compounds (words with meanings unrelated to those of their components, e.g. 馬上 literally horse-above 'immediately') did not facilitate response to semantically transparent targets sharing one of the characters (e.g. 馬掌 horse-palm' 'horseshoe'). Zhou and Marslen-Wilson [29] and Zhou et al. [31] found the strongest priming effects between compound words having a common morpheme (e.g. 華麗 *hua2 li4*[3] 'magnificent' and 華貴 *hua2 gui4* 'luxurious'), followed by words with homophonic and homographic morphemes (e.g. 華麗 *hua2 li4* 'magnificent' and 華僑 *hua2 qiao2* 'overseas Chinese') as well as compounds that are only related semantically with no phonological or orthographic overlap (e.g. 醫生 *yi1 sheng0* 'doctor' and 護士 *hu4 shi0* 'nurse'). Furthermore, there was no priming between lexical items with heterographic homophonic morphemes (e.g. 華麗 *hua2 li4* 'magnificent' and 滑翔 *hua2 xiang2* 'to glide').

The structure of the foregoing models with three interconnected levels of representations in effect suggests that reading aloud can be achieved via the "semantic" route, along which activation flows from orthographic representations to semantic features to phonological representations, or the "non-semantic" (or direct) route with direct access from orthographic to phonological units. This dual-route model of reading is corroborated

[1]In this paper, phonetic transcriptions are given in *jyutping*, a romanisation system developed by the Linguistics Society of Hong Kong. The tone of a syllable is represented by the number in the transcription.

[2]The semantic and the two form levels are directly connected in the model of Zhou et al. [31] and Zhou and Marslen-Wilson [30]. On the other hand, in Taft et al. [19], they are linked via a set of modality-free lemmas, which may correspond to bound morphemes, free morphemes, or multimorphemic words. The lemmas emerge as a result of repeated co-occurrence between the same semantic information and the same form information.

[3]Examples from these studies are given in *pinyin*, a romanisation system representing Mandarin Chinese.

by case studies of brain-injured individuals with dissociation between reading aloud and naming. Weekes, Chen, and Yin [24], Weekes and Chen [23], and Law and Or [9] have reported Mandarin- and Cantonese-speaking brain-damaged individuals who exhibit superior performance on reading to oral naming. The dissociation may be due to semantic deficits and/or disrupted access from semantics to phonology. Reading without semantics is further supported by the occurrence of a type of reading errors by these speakers, the legitimate alternative reading of components (or LARC errors [23]) first described in [13] for errors in reading Japanese Kanji characters.

The common form of LARC errors is reading aloud a pronounceable component in the target character [9, 23,24]. Some examples are given in (1) and we refer to them as RCC errors ("reading a character component").[4] The target characters in the example are phonetic compounds containing a semantic radical providing a cue to the meaning of the character and a phonetic radical providing a cue to the pronunciation of the character. For (1)b and (1)c, both radicals are pronounceable. Production of either constituent constitutes a RCC error, although dyslexic readers are far more likely to read aloud the phonetic radical, i.e., 野 and 乍.

(1)a. 挽 *waan5* –> 免 *min5*

b. 墅 *seoi6* –> 野 *je5* or 土 *tou2*

c. 昨 *zok6* –> 乍 *zaa3* or 日 *jat6*

Recently, another form of LARC errors was reported of a patient, LKK [7] involving target characters associated with different pronunciations in different word contexts, i.e. homographic heterophones, as illustrated in (2). Such errors are, henceforth, referred to as ROC errors ("reading out of context").

(2)a. 畫筆 'paintbrush' *waa2 bat1* –> *waak6 bat1* where 畫圖 *waak6 tou4* 'draw a picture'

b. 雪茄 'cigar' *syut3 gaa1* –> *syut1 ke2* where 蕃茄 *faan1 ke2* 'tomato'

c. 士巴啦 'spanner' *si6 baa1 laa2* –> *si6 baa1 laa3* where 啦 *laa3* is a sentence-final particle

While the presence of homographic heterophones in the lexicon would seem to require whole-word representations at the phonological level, at least for multisyllabic words containing them as suggested by Taft et al. [19], the production of ROC errors are in fact predicted by models with phonological representations corresponding to single syllables only. Characters with ambiguous pronunciations will map onto more than one syllable unit. In the absence of input from the semantic system to the phonological lexicon, the phonological representation inappropriate for the target word context may sometimes be selected for production, resulting in ROC errors. In other words, correct reading of homographic heterophones must involve semantic information in a system that contains only single syllable units. Take the lexical item in (2)b as an example. The target characters will independently make contact with the relevant syllable units via the non-semantic reading route, and access the semantic features associated with each of the characters. Crucially, the two characters will converge on a set of semantic units corresponding to the compound word, which will then activate the context-appropriate phonological units. This situation is illustrated in Fig. 1.

To examine the issue of whole-word phonological representations, one would need to study the reading of homographic heterophones of a speaker who performs at (near-)normal level in reading despite semantic deficits. Although LKK produced ROC errors, he is not suitable for such an investigation. Law argues that LKK suffers damage to the phonological lexicon and/or the access to it along the direct reading route and the semantic reading route at the post-semantic level, based on his near normal performance on comprehension tasks and impaired performance on all spoken tasks including naming, reading aloud, and word repetition [7]. In this paper, we present the results of a reading task with test items containing homographic heterophones and a comprehension task involving synonymy judgments from a Cantonese-speaking brain-injured dyslexic individual with a dissociation between impaired oral naming and preserved reading aloud performance [10]. His good performance on reading these words in contrast with his poor comprehension of them provides support for phonological representations corresponding to whole words. In addition, theoretical arguments for whole-word representations of all disyllabic and multisyllabic Chinese words are considered.

## 2. Case report

### *2.1. Subjects*

YKM was a 61-year-old right-handed male speaker of Cantonese. He was born in Mainland China and ob-

[4] Phonetic transcriptions to the left and to the right of the arrow represent target syllables and a subject's responses, respectively.

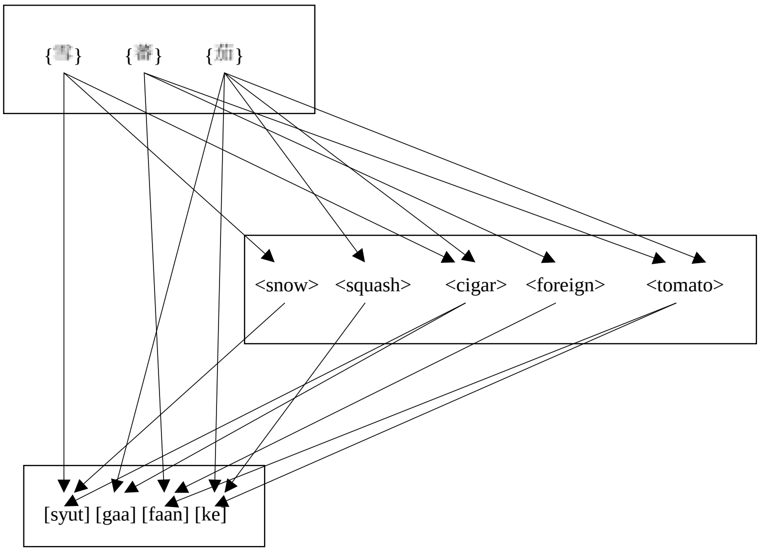

Fig. 1. Note. The symbols { }, < >, and [ ] stand for orthographic representations, semantic features, and phonological representations, respectively.

tained a university degree in Sociology in Hong Kong. He worked as a broker until the age of 50 when he suffered sub-arachnoid haemorrhage at the anterior communicating artery with arteriovenous clipping done. He then stayed in a medical rehabilitation center for three months with no speech or language therapy. In January 2002, he was admitted to the hospital with left basal ganglion haemorrhage. CT scans showed left intracerebral haematomas with small left frontal effusion and a small right cerebral infarct while no arteriovenous malformation aneurysm was detected. He had right-sided hemiparesis of the limbs and was diagnosed to have hypertension and cataract. His speech was non-fluent with mild dysarthria of the lips. His daily activity was reading the newspaper, but he often could not tell others what he had read.

YKM was previously assessed on reading aloud of single words, naming pictured objects, and comprehension of verbal and non-verbal materials [10]. His performance across these tasks is given in Table 1. His ability to name objects was clearly impaired. In contrast, he was able to read aloud the names of many of the objects he couldn't name, as well as single words of different frequencies of occurrence, grammatical word classes, and degrees of concreteness. He performed at below-normal level on all verbal and non-verbal comprehension tests. Hence, the dissociation of his impaired naming and preserved reading is argued to be due to semantic deficits vis-à-vis the largely intact direct reading route.

Four control subjects, one female and three males, with ages ranging from 35 to 63 years and more than 13 years of education were tested. Two of them were matched in age, education, and gender with YKM. Background information on the control subjects is given in Table 2.

### 2.2. Tasks and materials

Two tasks were administered to YKM, reading aloud and synonymy judgment. The same set of disyllabic words[5] containing homographic heterophones was used for both tasks. To construct this set of stimuli, we first identified 42 target characters with more than one pronunciation. According to the judgments of the two age- and education-matched controls, which were made after completion of the reading and comprehension tasks, 35 of them are associated with two phonological forms and seven with three possible pronunciations. For each of these characters, two words were chosen such that the same character is pronounced differently in these word contexts (e.g. 從容 *sung1 jung4* and 從新 *cung4 san1*). The 84 test items are mostly low word frequency words [25]. They range between 1 and 34 with a mean frequency of 5.7. Effort was made to ensure that members of each pair have comparable frequencies. The differences in frequency between words in each pair range from 0 to 15 with a mean difference of 3.8. As for other characteristics of the stimuli, six

[5] One exception was the morpheme 打 *daa1* 'dozen', for which the context was a trisyllabic phrase 一打蛋 'a dozen of eggs'.

Table 1
YKM's performance on confrontation naming, reading aloud, and semantic tasks [10]

| Task | Accuracy |
|---|---|
| Oral picture naming [16] ($n = 217$) | 72/217 (33.2%) |
| Reading names of objects [16] (1980) ($n = 217$) | 206/217 (94.9%) |
| Reading aloud single words [8] ($n = 390$) | 370/390 (94.9%) |
| Semantic tests | |
| Spoken word-picture matching ($n = 126$) | 102/126 (80.9%) |
| Written word-picture matching ($n = 126$) | 98/126 (77.8%) |
| Associative match task [14] ($n = 23$) | 15/23 (65.2%) |
| Pyramids and Palm Trees Test [6] ($n = 37$) | 22/37 (59.5%) |
| Naming by definition ($n = 216$) | 68/216 (31.5%) |
| Written version of Pyramids and Palm Trees Test ($n = 29$) | 15/29 (51.7%) |
| Synonymy judgment ($n = 60$)[a] | 43/60 (71.7%) |

[a]YKM would score one point for each correct response, i.e., acceptance of a synonymous pair or rejection of non-synonymous items.

Table 2
Background information on control subjects

| | EY | RW | LCS | KTF |
|---|---|---|---|---|
| Gender | F | M | M | M |
| Age | 37 | 44 | 64 | 62 |
| Education | University graduate | Post-secondary diploma | Postgraduate | University graduate |
| Dialect spoken at home | Cantonese | Cantonese | Cantonese | Cantonese |
| Occupation | Research assistant | IT technician | Part-time university lecturer | Director of a consultancy firm |
| Performance on the reading task | Not tested | Not tested | 84/84 | 84/84 |
| Performance on the synonymy judgment task | 82/84 | 84/84 | 84/84 | 84/84 |

words can be considered semantically opaque, e.g. 馬虎 *maa1 fu1* horse-tiger 'sloppy', 燒賣 *siu1 maai2* burn-sell 'a kind of dim sum'. In 13 of the 42 word pairs, the homographic heterophonic morphemes are semantically related (e.g. 平淡 *ping4 daam6* 'bland' and 鹹淡 *haam4 taam5* salty-tasteless 'taste'), whereas the others are unrelated (e.g. 交易 *gaau1 jik6* 'trading' and 輕易 *hing1 ji6* 'easy'). In terms of form class, there were 10 pairs with two nouns, seven pairs with two verbs, and the rest had words of different grammatical classes. As for the phonological difference between the pronunciations associated with the same target character, there were 18 cases where the two differ in tone only, another 10 instances where the difference involve the tone and the onset. Other forms of contrast include the whole syllable, the rime, the nucleus, the onset, and combinations of tone and a segmental. Finally, the target character occupies the first position in both words in 17 pairs, the second position in 12 pairs, and different positions in 13 pairs.

YKM was asked to read aloud the 84 stimuli once over two test sessions, with members of the same word pair being presented in separate sessions. A response was considered correct if the subject read aloud all the characters in the word. The two age- and education-matched controls performed flawlessly on these items.

For the synonymy judgment task, each test item was paired with a synonym and an unrelated word. On each of the 168 trials, the subject was presented with two written words, one containing a homographic heterophone and one that was either synonymous with or semantically unrelated to the target word. The subject would score one point if s/he correctly accepts the synonym and rejects the unrelated word. Three of the four control subjects were 100% correct, and the other rejected two synonyms.

### 2.3. Results

YKM read aloud 72/84 (85.7%) of test items and both members in 31/42 word pairs. All 12 errors were made on homographic heterophonic characters, nine of them were contextually inappropriate or ROC errors (e.g. 便宜 *pin4 ji4* 'cheap' –> *bin6 ji4* where 便利 *bin6 lei6* 'convenient'). The others included one semantic error, one phonological error, and one ambiguous error. Among the nine ROC errors, only one differed from the target in tone only; the others involved a difference in a segmental or tone and segmental.

In contrast, he only scored 44/84 (52.4%) in the synonymy judgment task. Of the 40 errors, 30 were acceptance of the synonym and the unrelated distractor; 9 involved rejections of both words, and one case with incorrect decisions on both trials.

Finally, there did not seem to be a relationship between YKM's abilities to read aloud and to comprehend a word. He could not comprehend 35/72 words he correctly read aloud, and for the 12 stimuli he misread, he showed some degree of understanding of the words in seven cases. It is also noted that the 35 words with correct production but no evidence of comprehension covered 30 different target characters, two of the six semantically opaque words, and included 19 nouns, eight verbs, seven adjectives, and one function word. In addition, the homographic heterophonic characters occupied the first position of the words in 19 cases and the second position in 16 instances.

## 3. Discussion

YKM exhibited a dissociation in performance between reading aloud and a task that is essentially supported by the semantic system. The findings are consistent with results of previous assessments that YKM demonstrated near normal ability to read aloud many words despite evidence of semantic deficits [10]. They show that his correct reading of characters with phonological forms that can solely be disambiguated in word contexts is independent of his comprehension of them. This is incompatible with the view that recognizes phonological representations of single syllables only. In such models, correct reading of homographic heterophones must involve semantic input. On the other hand, YKM's performance can be accounted for by models that assume the existence of whole-word phonological representations. In reading aloud, characters of a disyllabic or multisyllabic word independently access their syllable units; at the same time, the characters converge on the whole-word unit. As long as the direct reading route is intact, regardless of the functioning of the semantic reading route, the whole-word representation will be the most activated unit and therefore be selected for production.

The access of whole-word and syllable units by disyllabic words also explains the occurrence of ROC errors. These errors are produced when the target whole-word representation is for some reason unavailable, the syllable units corresponding to the possible pronunciations of the homographic heterophonic character will then compete for production, and the unit with higher level of activation or lower threshold will be more likely to be chosen. One factor that affects level of activation or threshold is frequency. In other words, ROC errors will be more likely to occur if the context inappropriate syllable is of higher frequency than the target syllable. This prediction seems to find some support from our data. For the nine ROC errors produced by YKM, the offending syllable has a higher frequency[6] than the target in six instances. This is so for both syllable frequency (based on a spoken Cantonese database [11]) and morpheme frequency [25].

It is noted that the claim for phonological representations of disyllabic and multisyllabic words also predicts a higher likelihood of ROC errors in reading aloud than oral naming. In the former task, the syllable unit inappropriate for the target word context will always be activated and compete with the target syllable. In the latter task, the contextually irrelevant unit will enter the competition only if it is associated with a morpheme semantically related to the target morpheme, such as *daam6* or *taam5* in 平淡 *ping4 daam6* 'bland' and 鹹淡 *haam4 taam5* salty-tasteless 'taste'. In summary, we propose that the phonological lexicon consists of whole-word and single syllable representations, similar to earlier models of the Chinese lexicon (e.g. [20,32]) but different from them in that these units are represented at the same level rather than in a hierarchical relationship.

Nevertheless, whole-word representations may only be required for a relatively small number of words, including those with homographic heterophones and monomorphemic disyllabic words such as binding words. Support for the latter comes from longer naming latencies of the second characters in binding words than those of the first characters [20]. Whole-word representations are not necessary for words with unique mapping between orthographic and phonological units. However, as pointed out in Taft et al. [19], information on the order of constituents within disyllabic or multisyllabic words must be represented in some way in the lexicon. Otherwise, we would not be able to recognize 馬掌 'horseshoe' and 頭痛 'headache' as real words and reject 掌馬 and 痛頭 as non-words, or to tell the difference in meaning between 牙刷 'toothbrush' and 刷牙 'to brush teeth', 傷風 'influenza' and 風傷 'wind erosion', or 眼紅 'jealous' and 紅眼 'red eye'. More-

[6] An arbitrary difference of at least 10 was used to categorize whether two units were different in frequency.

over, the relationship between morphemes of a compound cannot predict the order of the components. Although many morphologically complex words are of the type 'modifier-modified' (e.g. *pei4 haai4* leather-shoe 'leather shoes'), there are compounds in which the concept being modified precedes the modifier (e.g. *sam1 gap1* heart-hurry 'anxious, *daa2 dai1* hit-low 'knock down'), or the two components are of equal importance such as coordinate compounds (e.g. *wun2 dip6* bowl-dish 'china', *sau2 goek3* hand-foot 'limbs', *hoi1 gwaan1* open-close 'switch'). Reversing the order of the constituents of coordinate compounds will result in non-words. In short, the representation of the ordering of elements in all disyllabic words is necessary. The existence of whole-word phonological representations seems to be a straightforward solution.[7] Note that although an alternative account, analogous to the morpheme network approach, where syllable units are connected if they form a real word may also explain reading aloud of homographic heterophones, it remains unclear how information on the sequencing of syllables in a word is specified in such a network.

At present, the sequence of constituents in a disyllabic or two-character word is represented by connections marked for order information between lemmas and form units in Taft et al. [19], and by syllable and orthographic units that are individually specified for position of occurrence in Zhou et al. [31]. Neither treatment seems satisfactory. In a connectionist model, activation simply flows from one unit to the next. It is silent on how ordinal information can be incorporated in the mechanism. As for units with explicit marking of word position, given the fact that most morphemes can occupy various positions, units of identical phonological or orthographic content would have to be represented multiple times in the phonological and orthographic lexicons, respectively, depending on how many different positions they may occur in a word. If the move to do away with whole-word form representations was to reduce redundancy at these levels, it is not immediately clear how this approach is necessarily superior to the assumption of whole-word representations.

Further support for whole-word phonological representations can be drawn from another Cantonese dyslexic patient with similar deficits, CML [9]. In addition to the characteristic pattern of dissociation between better reading aloud than oral naming performance, CML made tonal errors (59% of errors) in reading far more frequently than she did in naming (4%). The researchers argue that the discrepancy can be explained if one makes the assumption about the presence of whole-word and syllable representations at the phonological level. Take 較剪 *gaau3 zin2* literally compare-cut 'scissors' as an example. When the characters are presented, the phonological units of *gaau3 zin2*, *gaau3*, and *zin2* will be accessed via the non-semantic reading pathway, and semantic features 'tool', 'sharp edges', 'to cut', 'made of steel', 'to compare' etc. will be activated via the semantic reading route, which then access *gaau3 zin2* 'scissors', dou1 'knife', goe3 'saw', *co3* 'file', *zin2* 'to cut', *gaau3* 'to compare' etc. In the event that the target entry *gaau3 zin2* and the syllable *gaau3* are deformed to become **gaau zin2* and **gaau*, respectively, a unit most closely resembles the target syllable, such as *gaau1* or *gaau2*, may be chosen for production, resulting in tonal errors **gaau1 zin2* or **gaau2 zin2*. In contrast, there is only semantic input to the phonological lexicon in a naming task; therefore, *gaau3 zin2*, *dou1*, *goe3*, *co3*, *zin2* will be activated. If the target disyllabic unit is unavailable, one of the other phonological representations may be selected, leading to a semantic error. The crucial difference between the two tasks is that the subject may circumvent the situation of degradation of target phonological entries by juxtaposing two syllable units independently accessed by the stimulus characters in reading aloud. Such an option is not always possible in naming, as the meaning of a compound word can rarely be derived directly and fully from the meanings of its constituent morphemes. If the subject produces a response through combining morphemes related to key semantic features of the object to be named, the output will likely be a nonword (e.g., *jyun4zi2 bat1* atom-pen 'ball pen' –> **se2 bat1* write-pen). Alternatively, the system may search for a disyllabic unit maximally similar to the target, but given that few semantically related multisyllabic words differ only in tone, tonal errors seldom occur in naming.

Finally, a phonological lexicon consisting of both whole-word and single syllable units makes certain interesting predictions about patterns of reading errors. A

[7] Recently, there are independent linguistic (albeit indirect) arguments for the existence of whole-word phonological representations of disyllabic lexical items in explaining the well-formedness of compounds composed of two or more words [4,5]. These accounts are prosodic or metrical in nature. For instance, Feng puts forth the notion of PrWd (prosodic word)-compound where its left edge must not break up any of its constituent words. Duanmu accounts for the preference for different lengths (monosyllabic or disyllabic) of words with practically identical meanings (e.g., *pin3* and *hei1pin3* 'to cheat') by both metrical structure and syntactic position. Moreover, metrical structure explains the internal ordering of words in [V-O N] and [M M N] compounds, where V, O, M, and N represent verb, object, modifier, and head noun, respectively.

character (associated with a particular morpheme) may appear in different word contexts, e.g. *faan4* "numerous" in *faan4 sing1* "an array of stars" or faan4 man4 "detailed forms". If these words are independently represented at the phonological level, it is possible that a dyslexic individual may correctly name the character in one context but not the other. Such dissociation may be more easily observed in individuals whose performance is sensitive to psycholinguistic variables such as word frequency. In other words, the same character may be more likely to be read aloud when it is in a high frequency word than a low frequency lexical item. Similarly, a dyslexic speaker may be able to read aloud a character presented alone but unable to do so when it occurs in a word, or vice versa. These patterns of performance are not predicted by a lexicon containing only syllable units. In addition, referring to the case of CML discussed earlier, a proper evaluation of the explanation for the difference in frequency of tonal errors between oral naming and reading aloud can be carried out through contrasting the rates of tonal errors in the two tasks as a function of word length. The prediction is that discrepancies are only observable in disyllabic and multisyllabic words but not monosyllabic items, if the proposed account is correct. This is because few disyllabic/multisyllabic words differ only in tone compared with monosyllabic lexical items. Recently, naming latencies for homophones (e.g., [n ∧ n]) have been found to be affected by specific-word frequency (i.e., *nun*) rather than cumulative-homophone frequency (i.e., frequency of *nun* and *none*) from a picture naming task in English and Mandarin Chinese, and a task translating Spanish into English [2]. In that study, the Chinese stimuli were all monosyllabic. According to Milsky (1974) (as cited in Wang [22]), about 10% of multisyllabic words in Mandarin Chinese are homophonous. The relevance of this estimate and the findings in Caramazza et al. to the issue in the present study is that if multisyllabic words are represented phonologically as whole units, naming response times for multisyllabic homophones should be best predicted by target word frequency as opposed to cumulative-homophone frequency, frequencies of the constituent morphemes, or cumulative frequencies of homophonous morphemes.

## 4. Conclusion

This paper has described the performance of a Cantonese dyslexic patient, YKM, on reading aloud and comprehension of disyllabic words containing homographic heterophonous characters. YKM correctly produced many stimuli that he could not understand. This finding together with the occurrence of ROC errors favors the claim for representations of both single syllables and whole words in the Chinese lexicon. If whole-word phonological representations exist in a language that has the characteristics of (i) little inflectional morphology, (ii) unique mapping from orthography to phonology for the great majority of cases, and (iii) morphologically complex words, which are mostly compounds, are simple concatenations of the phonological forms of their constituent morphemes, it seems reasonable that their presence in the mental lexicon is a feature of all languages.

## Acknowledgements

The work reported here was supported by a grant from the Research Grant Council of Hong Kong (HKU 7157/02H). We are grateful to YKM for his participation in this study.
